# Coordination-driven self-assembly of discrete Ru_6_–Pt_6_ prismatic cages

**DOI:** 10.3762/bjoc.14.199

**Published:** 2018-08-27

**Authors:** Aderonke Ajibola Adeyemo, Partha Sarathi Mukherjee

**Affiliations:** 1 Department of Inorganic and Physical Chemistry, Indian Institute of Science, Bangalore 560 012, India

**Keywords:** arene–ruthenium(II), heterometallic cages, platinum metalloligand, self-assembly, supramolecular architectures

## Abstract

The coordination-driven self-assembly of two new Ru_6_–Pt_6_ hexanuclear trigonal prismatic cages comprising arene–ruthenium(II) clips (**1a(NO_3_)_2_** and **1b(NO_3_)_2_**) and a tritopic platinum(II) metalloligand **2** has been performed in methanol at room temperature. The [3 + 2] hexanuclear cages **3a** and **3b** were isolated in good yields and characterized by well-known spectroscopic techniques including multinuclear NMR, mass spectrometry, UV–vis and infrared studies. Geometry optimization revealed the shapes and sizes of these hexanuclear prismatic cages. The combination of ruthenium and platinum metal center in a one-pot self-assembly reaction showcases the construction of aesthetically elegant heterometallic structures in supramolecular chemistry leading to the formation of a single major product.

## Introduction

Coordination-driven self-assembly of discrete architectures has evolved as a unique protocol to construct elegant supramolecular architectures of different shapes, sizes and functionalities over the last two decades [1–23]. These 2D and 3D-supramolecular architectures mostly comprise pure organic ligands as electron-rich donors and transition metals as electron-deficient acceptors. Diverse functionalities embedded in these homometallic architectures have found useful applications in chemical sensing [24–35], catalysis [11,36–46], drug delivery [47–51] and host–guest chemistry [52–56] among others. The cardinal prerequisites to obtain these self-assembled supramolecular architectures include stoichiometry and conformational complementarity on the binding sites of the building blocks [57–61]. However, the use of a single metal and a single organic ligand design may limit the structural diversity as well as the functionality of homometallic supramolecular architectures. In the last few years, enormous efforts have been channeled towards multicomponent self-assembly involving the construction of sophisticated heterobimetallic supramolecular architectures in a one-pot reaction and their functional properties are currently being explored [55,62–70]. The incorporation of two different metal centers in a supramolecular architecture can impart different functional properties arising from each of the metals and/or the organic components which is quite interesting.

The metalloligand synthetic approach has been efficiently used to achieve the construction of heterobimetallic supramolecular architectures in order to minimize the formation of two independent homometallic architectures or a mixture of products [71–78]. The metalloligand is a kinetically stable coordination complex with a metal center and one or more appended donor site(s) which can further coordinate to another metal center [79–83]. Such metalloligands are predesigned and encrypted with the desired functional properties before they are used in self-assembly reactions so as to induce the desired functional properties into the final supramolecular architecture [84–92]. Hence, the metalloligand becomes the electron-rich building block which offers structural rigidity while the second metal is the electron-acceptor building block. The facile self-assembly of 2D-heterobimetallic supramolecular architectures are well reported in the literature [24,93–94], however, 3D-heterobimetallic systems are still less explored [95–97].

**Scheme 1:**
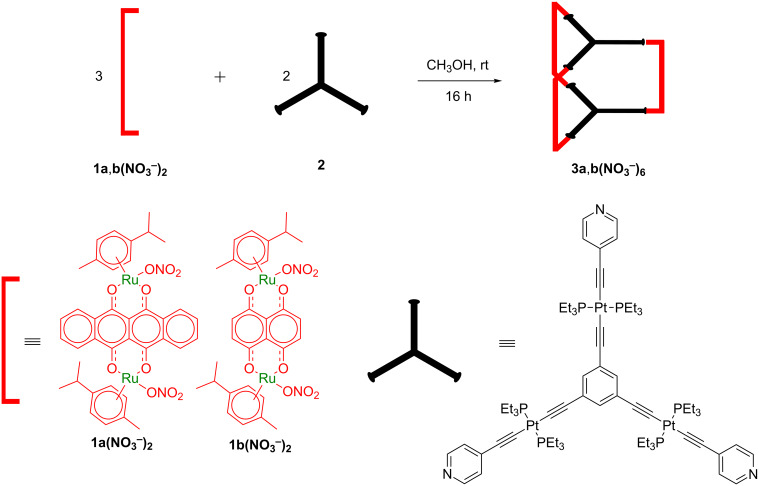
Self-assembly of the heterometallic prismatic cages.

Dinuclear arene–ruthenium(II) acceptor clips/building blocks have been extensively utilized in supramolecular chemistry because of their rigid directionality toward electron-rich donors due to their restricted coordination sites as a result of the fixed position of the *p*-cymene moiety [24,93,98–103]. Our group and others have contributed substantially to the chemistry of self-assembled homometallic ruthenium architectures and their applications [100,102–108]. In broadening this research scope, herein, we describe the coordination-driven self-assembly of two new Ru–Pt heterometallic prismatic cages **3a** and **3b** obtained from the reaction of two arene–ruthenium(II) clips **1a** and **1b** and tritopic platinum(II) metalloligand **2** in methanol/chloroform mixture in 3:2 ratio (Scheme 1). Both cages were fully characterized by ^1^H, ^31^P, ^195^Pt, ^1^H,^1^H COSY, DOSY NMR, electrospray ionization mass spectrometry, and UV–vis analysis. Further structural insights were revealed by computational studies.

## Results and Discussion

**Scheme 2:**
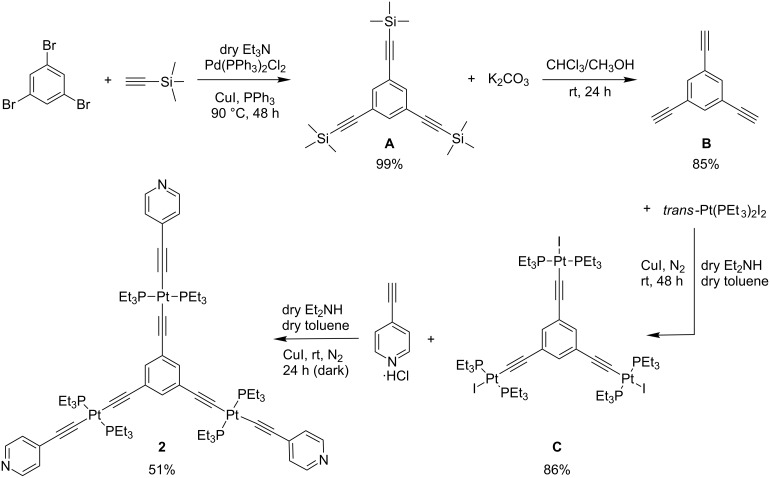
Synthesis of the platinum metalloligand **2**.

The triplatinum metalloligand **2** was synthesized through a four-step reaction involving a Sonogashira coupling (Scheme 2) and the crude product was purified by column chromatography to obtain **2** as a yellow powder. The three intermediates **A**, **B** and **C** were also characterized by ^1^H, ^31^P, ^195^Pt and ^13^C NMR analyses (see Supporting Information File 1, Figures S1–S8). The metalloligand **2** is highly soluble in dichloromethane and chloroform but only partially soluble in methanol and acetonitrile. The ^1^H, ^31^P, ^195^Pt and ^13^C NMR experiments (Supporting Information File 1, Figures S9–S12) and electrospray ionization mass spectrometry (ESIMS, Supporting Information File 1, Figure S13) of metalloligand **2** evidenced the formation of a pure compound. The ^1^H NMR spectrum of **2** revealed two doublets and a singlet in the downfield region (8.38–6.99 ppm) corresponding to the pyridyl protons and the central phenyl protons, respectively, while the methylene and methyl protons in the upfield region of the spectrum are observed between 2.16–1.18 ppm (Supporting Information File 1, Figure S9). The ^31^P NMR spectrum of **2** gave a singlet peak at 11.18 ppm shifting downfield after coordination with the ethynylpyridine moiety, while the singlet ^195^Pt peak remained almost the same with the precursor compound **C** (Supporting Information File 1, Figure S12). The mass spectrum of **2** shows a [**2** + H]^+^ peak at *m*/*z* 1748.59 (Supporting Information File 1, Figure S13) which is in good agreement with the calculated value of 1748.71 based on the C_69_H_105_N_3_P_6_Pt_3_ molecular formula.

### Self-assembly and characterization of the heterometallic cages

The treatment of the dichloride analogues of **1a** and **1b** with 2.1 equivalents of silver nitrate in methanol at room temperature for 3 hours gave the dinuclear arene–ruthenium(II) clips **1a**,**1b(NO_3_)_2_**. As represented in Scheme 1, the self-assembly reactions of methanolic solutions of **1a**,**1b(NO_3_)_2_** and methanolic solution of the triplatinum metalloligand **2** at room temperature yielded the trigonal prismatic cages **3a** and **3b**. The heterometallic prismatic cages were isolated as nitrate complexes in good yields. The isolated cages are soluble in methanol, acetonitrile, acetone, nitromethane, dimethyl sulfoxide and partially soluble in chloroform and dichloromethane. The formation of these cages was ascertained by multinuclear NMR experiments and ESIMS analyses.

**Figure 1:**
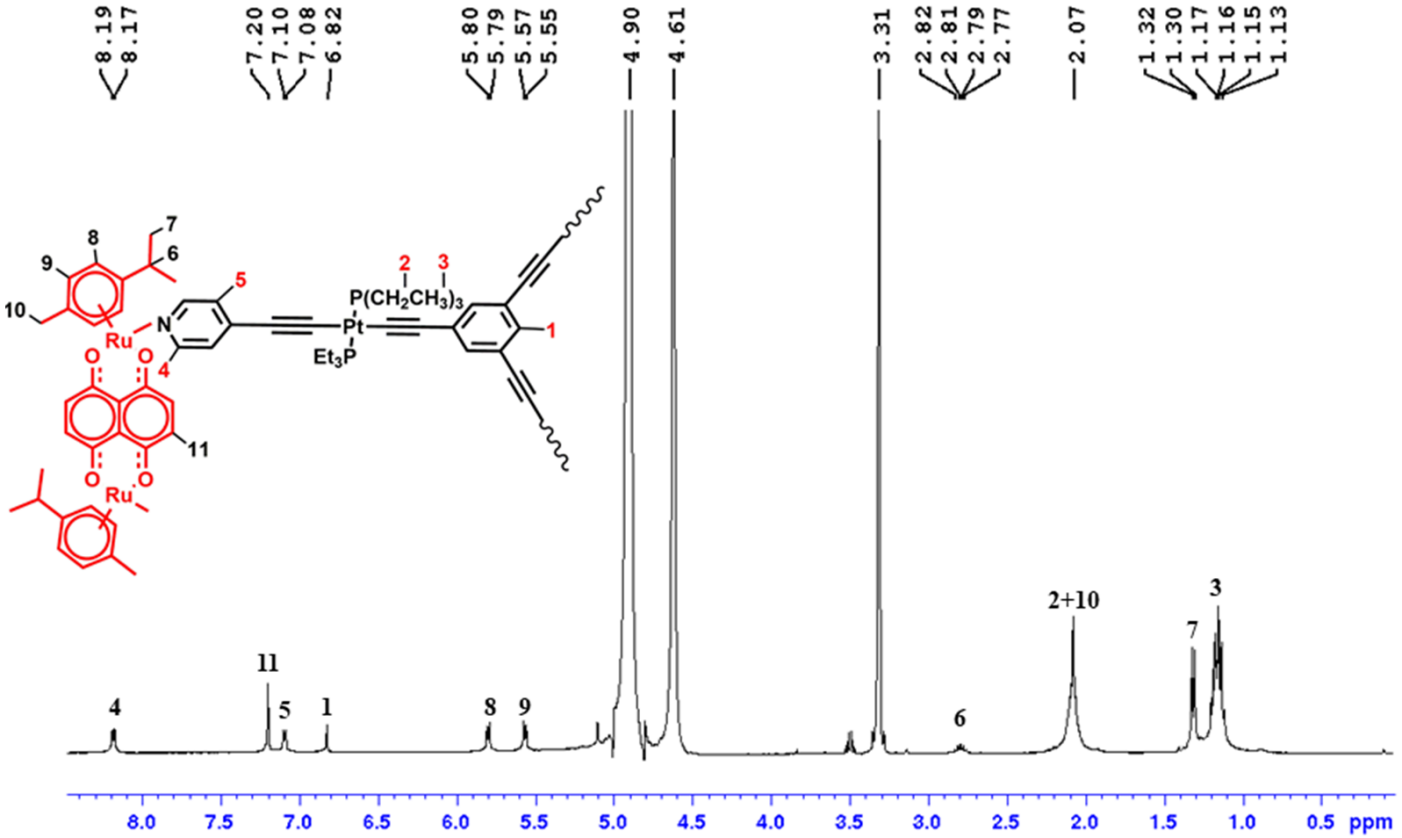
^1^H NMR of **3b** in CD_3_OD.

An upﬁeld shift was observed in the pyridyl protons of cage **3b** as compared to the free triplatinum metalloligand **2** in their ^1^H NMR spectra while cage **3a** exhibited a downfield shift in the pyridyl protons (Figure 1 and Supporting Information File 1, Figure S14). This chemical shift is due to the coordination of the pyridyl nitrogen atom to the ruthenium metal center while the upfield shift is due to the shielding effect of the methyl/methylene protons of the *p*-cymene moiety. In both cages studied, the aromatic protons of the *p*-cymene moiety in **3a** and **3b** were slightly shifted downfield while the isopropyl and methyl protons of the *p*-cymene moiety in all the cages remained almost unchanged as compared to the arene–ruthenium(II) clips **1a**,**1b(NO_3_)_2_**. Additionally, the protons of naphthacenedione in **3a** and naphthaquinone in **3b** were not considerably shifted (Figure 1 and Supporting Information File 1, Figure S14). The appearance of a singlet peak in the ^31^P NMR evidenced the formation of a single product and the fact that the phosphorus moieties are in the same chemical environment (Supporting Information File 1, Figures S15 and S16). The same observation is recorded for the ^195^Pt NMR analyses of all the heterometallic cages (Supporting Information File 1, Figures S17 and S18).

The DOSY NMR experiments also confirmed the formation of a single product in all the cages with the hydrodynamic radii (*r*_H_) of the heterometallic prismatic cages calculated from the Stokes–Einstein equation using the diffusion coefficients (*D*) obtained from the DOSY NMR experiments. The obtained values of *D* from the experiment are −9.631 log (m^2^ s^−1^) for **3a** and −9.567 log (m^2^ s^−1^) for **3b**, respectively. The calculated hydrodynamic radii (*r*_H_) of **3a** and **3b** are 15.57 Å, and 13.43 Å, respectively (Supporting Information File 1, Figures S19 and S20). The ^1^H,^1^H COSY NMR spectra also showed the correlation between the protons of the arene–ruthenium(II) clips as well as the correlation between the protons within the metalloligand (Supporting Information File 1, Figures S19 and S20).

The vibrational symmetrical stretching frequency of the coordinated carbonyl groups (ν_C–O_) in the dinuclear arene–ruthenium(II) clips **1a** and **1b** was found at 1536.16 cm^−1^ for **3a** and 1528.93 cm^−1^ for **3b** in the infrared spectra of the heterometallic cages while the vibrational symmetrical stretching frequency bands of =C–H_aromatic_ showed strong stretching bands at 3074.07 cm^−1^ for **3a** and 3064.78 cm^−1^ for **3b**, respectively. Additionally, the stretching bands at 543.67 cm^−1^ for **3a** and 545.44 cm^−1^ for **3b** correspond to the ν_Ru–O_ symmetrical stretching frequency (Supporting Information File 1, Figure S21).

**Figure 2:**
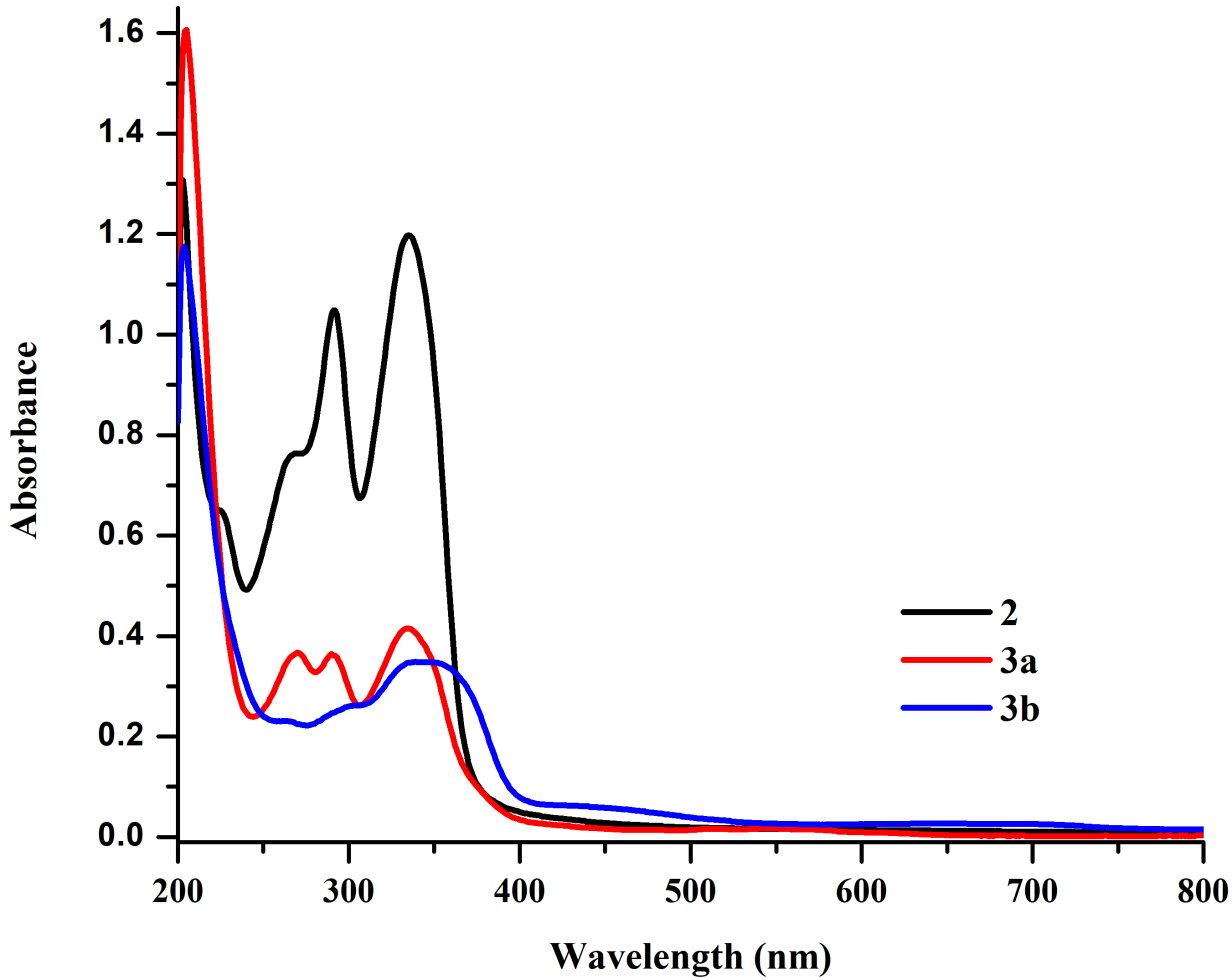
UV–vis spectra of the metalloligand **2** and heterometallic prismatic cages **3a** and **3b** in methanol (1.0 × 10^−5^ M) at 298 K.

The UV–vis absorption spectra recorded in methanol at room temperature show intense bands at λ_max_ = 544, 514, 334, 290, 205 nm for **3a** and λ_max_ = 698, 644, 339, 204 nm for **3b**. The intense bands at 335 nm and 291 nm for **2** correspond to charge-transfer transitions, which shift slightly to shorter wavelengths in the spectra of the heterometallic prismatic cages. The peaks in the ranges of 514–698 nm and 204–339 nm can be assigned to intramolecular and intermolecular π–π* transitions and metal-to-ligand charge transfer (MLCT) transitions associated with capped *p*-cymene ruthenium cap, respectively. A hypochromic shift (decrease in absorption intensity) was also observed in the spectra of the heterometallic prismatic cages as compared to the metalloligand probably as a result of coordination of electron-rich metalloligand to the electron-deficient ruthenium center (Figure 2).

**Figure 3:**
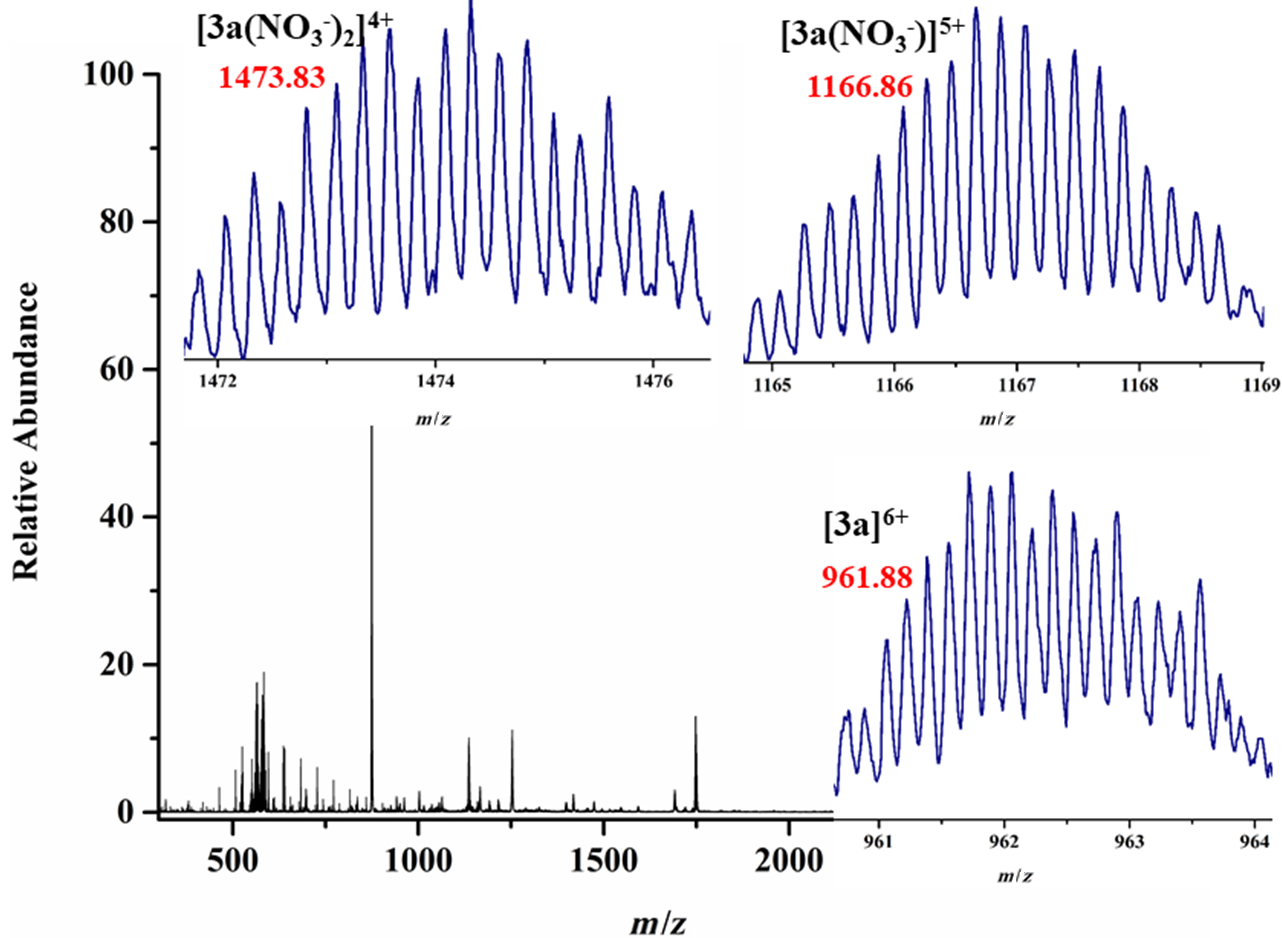
ESIMS spectrum of **3a** in methanol. Inset: experimentally observed isotopic distribution patterns of the charged fragments.

Mass spectrometry experiments also established the formation of the heterometallic prismatic cages in which all the cages maintain good stability. The ESIMS analysis of the [3 + 2] self-assembled heterometallic cages showed multiply charged fragmented ions for **3a** at *m/z* = 1473.83 [**3a**(NO_3_^−^)_2_]^4+^, 1166.86 [**3a**(NO_3_^−^)]^5+^, 961.88 [**3a**]^6+^; **3b** at *m/z* = 1398.79 [**3b**(NO_3_^−^)_2_]^4+^, 1106.64 [**3b**(NO_3_^−^)]^5+^, 911.87 [**3b**]^6+^ and all peaks are well-resolved isotopically and matched with the theoretical isotopic distribution patterns (Figure 3 and Supporting Information File 1, Figure S22).

### Geometry optimization of the heterometallic cages

**Figure 4:**
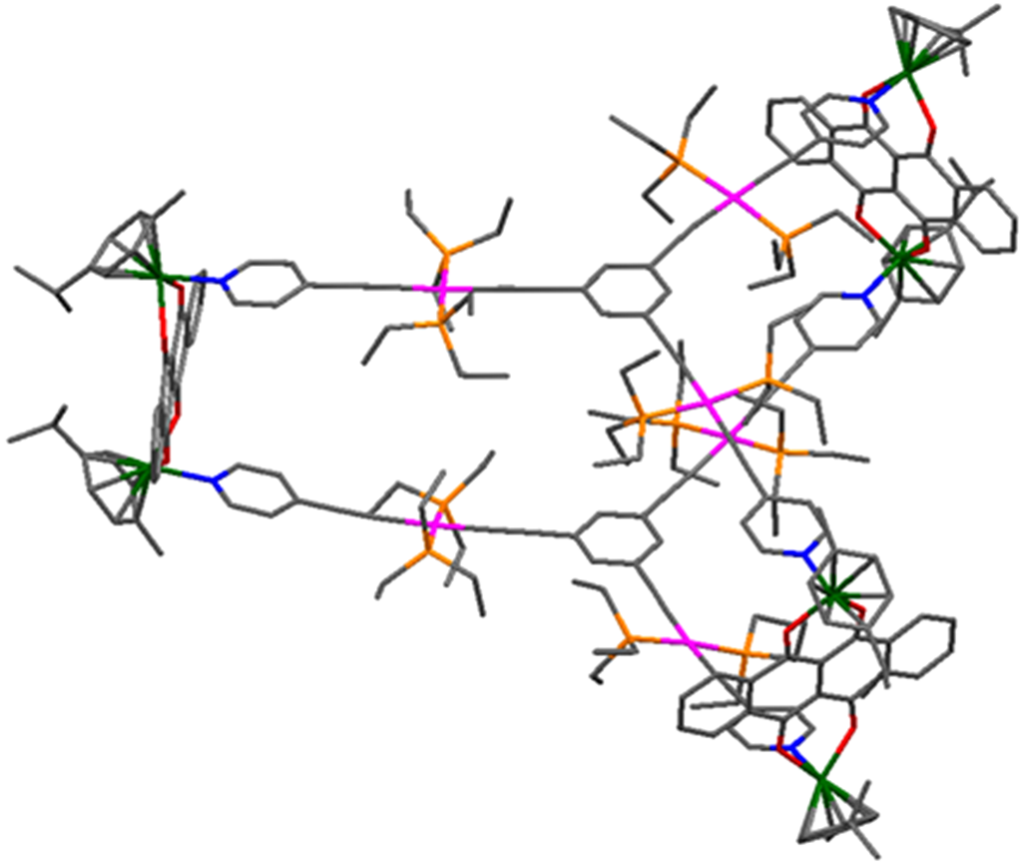
Energy-minimized structure of heterometallic trigonal prismatic cage **3a**. Hydrogen atoms are omitted for the sake of clarity [Ru: green, Pt: pink, O: red, N: blue, P: orange, C: grey].

All efforts to obtain single crystals of the prismatic cages were unsuccessful so far. Thus the structures of **3a** and **3b** were optimized to get insights into their structural features. The tritopic platinum(II) metalloligand was optimized using the B3LYP method while the heterometallic cage structures **3a** and **3b** were optimized with the semiempirical method using the PM6 basis set. The energy-minimized structures showed that cage **3a** has a dimension of 8.414 Å × 26.321 Å × 26.755 Å while cage **3b** has a dimension of 8.231 Å × 26.227 Å × 26.598 Å. The phenyl cores of the two triplatinum metalloligands are separated by a distance of 11.376 Å in **3a** and 11.456 Å in **3b**. The phenyl cores are slightly out-of-plane with regards to the pyridyl groups possibly as a result of steric influence upon metal–ligand coordination (Figure 4 and Supporting Information File 1, Figure S23).

## Conclusion

The metalloligand synthetic approach has been utilized to synthesize heterobimetallic trigonal prismatic coordination cages **3a** and **3b** through the one-pot coordination-driven self-assembly of dinuclear arene–ruthenium(II) clips and a tritopic platinum(II) metalloligand. The formations of the [3 + 2] trigonal prismatic Ru–Pt cages are confirmed by multinuclear NMR and ESIMS studies. The optimized structures of cages **3a** and **3b** showed large prismatic cages composed of twelve metal centers comprising six ruthenium(II) and six platinum(II) metal centers thus showcasing how two different metal components can be incorporated to form a single framework architecture through a one-pot self-assembly strategy.

## Supporting Information

File 1 Experimental procedures, multinuclear NMR spectra data, ESIMS data and infrared spectra of the hexanuclear trigonal prismatic cages.
