# Supplementary material for: Coordination-driven self-assembly of discrete Ru6–Pt6 prismatic cages
Source: Beilstein J Org Chem. 2018 Aug 27;14:2242–9. doi: 10.3762/bjoc.14.199 (PMC6122361; doi:10.3762/bjoc.14.199)
Supplement: File 1 — Experimental procedures, multinuclear NMR spectra data, ESIMS data and infrared spectra of the hexanuclear trigonal prismatic cages. [file Beilstein_J_Org_Chem-14-2242-s001.pdf]

Supporting Information

for

Coordination-driven self-assembly of discrete Ru_6–Pt_6 prismatic cages

Aderonke Ajibola Adeyemo and Partha Sarathi Mukherjee*§

Address: Department of Inorganic and Physical Chemistry, Indian Institute of Science, Bangalore-560 012, India

Email: Partha Sarathi Mukherjee - psm@iisc.ac.in

*****Corresponding author

§Tel.: +91-80-22933352; Fax: +91-80-23601552

Experimental procedures, multinuclear NMR spectra data, ESIMS data and infrared spectra of the hexanuclear trigonal prismatic cages

Experimental

General methods: The arene-ruthenium(II) clips $[Ru_2(dhtq)(H_2O)_2(\eta^6\text{-}p\text{-cymene})_2](NO_3)_2$ (**1a**) and $[Ru_2(dhnq)(H_2O)_2(\eta^6\text{-}p\text{-cymene})_2](NO_3)_2$ (**1b**) were prepared under dry nitrogen conditions using standard Schlenk techniques [1-2]. Methanol, diethylamine, toluene and triethylamine were dried and distilled under nitrogen according to standard literature

procedures. Silver nitrate, 1,3,5-tribromobenzene, ethynylpyridine hydrochloride, bis(triphenylphosphine)palladium(II) dichloride and K_2PtCl_4 were purchased from Alfa Aesar while triphenylphosphine, trimethylsilylacetylene and copper iodide were purchased from Sigma Aldrich and used without further purification. ^{1}H, ^{13}C, ^{31}P, ^{195}Pt, COSY and DOSY NMR spectra were recorded on a Bruker 400 MHz spectrometer. The chemical shifts (δ) in ^{1}H NMR spectra are reported in ppm with reference to $(CH_3)_4Si$ as internal standard at 0.0 ppm or to proton resonances resulting from incomplete deuteration of the NMR solvents as in CD_3OD at 3.31 and $CDCl_3$ at 7.26 ppm. ^{13}C NMR spectra were recorded at 100 MHz and the chemical shifts are reported in ppm with reference to external $CDCl_3$ at 77.84–77.20 ppm. Infrared spectra were recorded on a Bruker ALPHA FT-IR spectrometer. ESIMS data of the heterometallic cages were recorded in the positive mode using an Agilent 6538 Ultra-High Definition (UHD) Accurate Mass Q-TOF spectrometer in standard spectroscopic grade solvent (CH_3OH). UV–vis studies were carried out on a Shimadzu UV-2600 UV-Vis spectrophotometer in spectroscopic grade methanol using precision cells made of quartz (1 cm). Detailed NMR spectra and assignments for the hexanuclear cages are reported below.

Synthesis of triplatinum metalloligand 2

Compound A: Freshly dried triethylamine (50 mL) was distilled into a flame-dried 100 mL two-necked round-bottomed flask containing 1,3,5-tribromobenzene (2 g, 6.35 mmol), $Pd(PPh_3)_2Cl_2$ (223 mg, 0.05 mmol), PPh_3 (41.66 mg, 0.03 mmol) and CuI (48 mg, 0.04 mmol). The solution was refluxed at 50 °C for 15 min and trimethylsilylacetylene was added. The reaction mixture was refluxed for 48 h at 90 °C after which triethylamine was fully removed on a rotary evaporator. The black solid was chromatographed on a silica gel column using hexanes to give a light yellow powder as the expected product. Isolated yield: 2.35 g. ^{1}H NMR ($CDCl_3$, 400 MHz): δ (ppm) 7.49 (s, 3H, H_1), 0.24 (s, 27H, H_2). $^{13}C\{^{1}H\}$ NMR ($CDCl_3$, 100 MHz): δ (ppm) 135.35, 124.14, 103.62, 96.02, 0.31.

Compound B: **A** (1 g, 2.73 mmol) and K_2CO_3 (1.24 g, 8.99 mmol) were dissolved in 100 mL $CHCl_3$/MeOH 3:2 and stirred at rt for 24 h. The solvent was removed on a rotary evaporator and the off-white solid was purified by silica gel column chromatography using dichloromethane/hexane 1:4 to give the product. Isolated yield: 350 mg. ^{1}H NMR ($CDCl_3$, 400 MHz): δ (ppm) 7.57 (s, 3H, H_1), 3.11 (s, 3H, H_2). $^{13}C\{^1H\}$ NMR ($CDCl_3$, 100 MHz): δ (ppm) 136.12, 132.48, 123.38, 123.01, 83.51, 82.10, 70.59, 79.19.

Compound C: **B** (200 mg, 1.33 mmol), *trans*-Pt$(Et_3)_2I_2$ (3.65 g, 5.33 mmol) and CuI (38 mg, 0.2 mmol) were weighed into a flame-dried 100 mL Schlenk flask. Dry diethylamine (40 mL) was added into the Schlenk flask followed by 20 mL of dried toluene. The flask was degassed under vacuum and refilled with nitrogen three times. The solution was stirred at rt for 48 h. The orange solution was filtered to remove white solid of Et_2NH_2I and the filtrate evaporated on a rotary evaporator. The dirty-yellow solid was purified by silica gel column chromatography using hexane/ethyl acetate 1:1 to give an orange powder. Isolated yield: 2.1 g. ^{1}H NMR ($CDCl_3$, 400 MHz): δ (ppm) 7.01 (s, 3H, H_1), 2.23 - 2.18 (m, 36H, H_2), 1.17 - 1.13 (d, 54H, H_3). ^{31}P NMR ($CDCl_3$, 162 MHz): δ (ppm) 8.57. $^{13}C\{^1H\}$ NMR ($CDCl_3$, 100 MHz): δ (ppm) 130.63, 128.51, 100.35, 89.54, 30.15, 17.24, 17.07, 16.89, 8.77. ^{195}Pt NMR ($CDCl_3$, 86 MHz): δ (ppm) 291.14.

2: **C** (500 mg, 0.27 mmol) and 4-ethynylpyridine hydrochloride (153.21 mg, 1.10 mmol) were weighed into a flame-dried 100 mL Schlenk flask. Dried diethylamine (15 mL) was then distilled directly into the Schlenk flask followed by 15 mL of dried toluene. Copper(I) iodide (5.23 mg, 0.03 mmol) was added to the reaction mixture and the mixture was stirred in the dark at rt for 24 h. The solvent was removed under vacuum and the crude product was purified by column chromatography using dichloromethane/acetone 1:1 to 1:4 as the eluent to isolate the product. Isolated yield: 245 mg. ^{1}H NMR ($CDCl_3$, 400 MHz): δ (ppm) 8.38 - 8.37

(d, 6H, H_4), 7.09 - 7.07 (d, 6H, H_5), 6.99 (s, 3H, H_1), 2.16 - 2.12 (m, 36H, H_2), 1.22 - 1.18 (m, 54H, H_3). ^{31}P NMR ($CDCl_3$, 162 MHz): δ (ppm) 11.18. ^{13}C{^{1}H} NMR ($CDCl_3$, 100 MHz): δ (ppm) 149.60, 137.14, 131.03, 128.28, 125.87, 118.08, 117.94, 110.43, 108.13, 106.16, 106.01, 105.86, 32.38, 32.20, 30.15, 23.14, 16.82, 16.65, 14.57, 8.78, 6.20, 1.47. ^{195}Pt NMR ($CDCl_3$, 86 MHz): δ (ppm) 291.20. ESI-MS (*m/z*): Found: 1748.59 [**2** + H]$^+$; Calc'd: 1748.71 [**2** + H]$^+$. IR (neat): υ/cm^{-1} 3061.18 (s, =C-H$_{\text{-aromatic}}$), 1583.06 (s, C-C$_{\text{-aromatic}}$), 1482.11 (s, C-N$_{\text{pyridyl}}$). UV-Vis (CH_3OH) λ_{max} (ε) [nm (cm^{-1}M^{-1})]: 335 (9.6 x 10^4), 291 (8.4 x 10^4).

Coordination-driven self-assembly of the heterometallic cages: A methanol solution of three equivalents of **1a** and **1b** was stirred separately with the methanol solution of two equivalents of **2** in a closed 4 mL vial at rt for 16 h. An immediate visual color change showed the progress of the reactions. Each reaction mixture was filtered through a cotton plug and the solution was concentrated under reduced pressure to about 0.5 mL. The expected solids were precipitated in cold diethyl ether and dried under vacuum.

3a: Arene-ruthenium(II) clip **1a** (10 mg, 0.01 mmol) and 2 (13.58 mg, 0.01 mmol) were stirred in methanol at room temperature to obtain **3a**. Isolated yield: 80 %. ^{1}H NMR (CD_3OD, 400 MHz): δ (ppm) 8.69 - 8.68 (d, 12H, H_4), 8.34 - 8.32 (d, 12H, H_{12}), 7.94 - 7.93 (d, 12H, H_{11}), 7.18 - 7.17 (d, 12H, H_5), 6.90 (s, 6H, H_1), 5.96 - 5.95 (d, 12H, H_8), 5.73 - 5.71 (d, 12H, H_9), 2.91 - 2.86 (m, 6H, H_6), 2.20 - 2.18 (s, 18H, H_{10}), 2.04 - 1.99 (m, 72H, H_2), 1.34 - 1.04 (m, 144H, H_3 + H_7). ^{31}P NMR (CD_3OD, 162 MHz): δ 11.73 ppm. ^{195}Pt NMR (CD_3OD, 86 MHz): δ 291.14 ppm. ESI-MS (*m/z*): Calc'd: 1473.99 [**3a**(NO_3^-)$_2$]$^{4+}$, 1166.79 [**3a**(NO_3^-)]$^{5+}$, 961.99 [**3a**]$^{6+}$; Found: 1473.83 [**3a**(NO_3^-)$_2$]$^{4+}$, 1166.86 [**3a**(NO_3^-)]$^{5+}$, 961.88 [**3a**]$^{6+}$. IR (neat): υ/cm^{-1} 3074.07 (w, =C-H$_{\text{aromatic}}$), 1597.25 (s, C-C$_{\text{-aromatic}}$), 1536.16 (s, C-O), 543.67 (s, Ru-O). UV-Vis (CH_3OH) λ_{max} *(ε)* [nm (cm^{-1}M^{-1})]: 544 (1.7 x 10^3), 514 (1.6 x 10^3), 334 (4.2 x 10^4), 290 (3.6 x 10^4), 205 (1.6 x 10^5).

3b: Arene-ruthenium(II) clip **1b** (10 mg, 0.01 mmol) and **2** (15.40 mg, 0.01 mmol) were stirred in methanol at room temperature to obtain **3b**. Isolated yield: 86 %. ^{1}H NMR (CD_3OD, 400 MHz): δ (ppm) 8.19 - 8.17 (d, 12H, H_4), 7.20 (s, 12H, H_{11}), 7.10 - 7.08 (d, 12H, H_5), 6.82 (s, 6H, H_1), 5.80 - 5.79 (d, 12H, H_8), 5.57 - 5.55 (d, 12H, H_9), 2.82 - 2.77 (m, 6H, H_6), 2.07 (s, 90H, H_2 + H_{10}), 1.32 - 1.30 (d, 36H, H_7), 1.17 - 1.13 (m, 108H, H_3). ^{31}P NMR (CD_3OD, 162 MHz): δ 11.61 ppm. ^{195}Pt NMR (CD_3OD+$CDCl_3$, 86 MHz): δ 291.13 ppm. ESI-MS (*m/z*): Calc'd: 1398.90 $[3b(NO_3^-)_2]^{4+}$, 1106.72 $[3b(NO_3^-)]^{5+}$, 911.93 $[3b]^{6+}$; Found: 1398.79 $[3b(NO_3^-)_2]^{4+}$, 1106.64 $[3b(NO_3^-)]^{5+}$, 911.87 $[3b]^{6+}$. IR (neat): υ/cm^{-1} 3064.78 (w, =C-$H_{aromatic}$), 1564.24 (s, C-$C_{-aromatic}$), 1528.93 (s, C-O), 545.44 (s, Ru-O). UV-Vis (CH_3OH) λ_{max} (ε) [nm (cm^{-1}M^{-1})]: 698 (2.6 x 10^3), 644 (2.7 x 10^3), 339 (3.5 x 10^4), 204 (1.1 x 10^5).

Multinuclear NMR spectra data, ESIMS data and infrared spectra of the hexanuclear trigonal prismatic cages

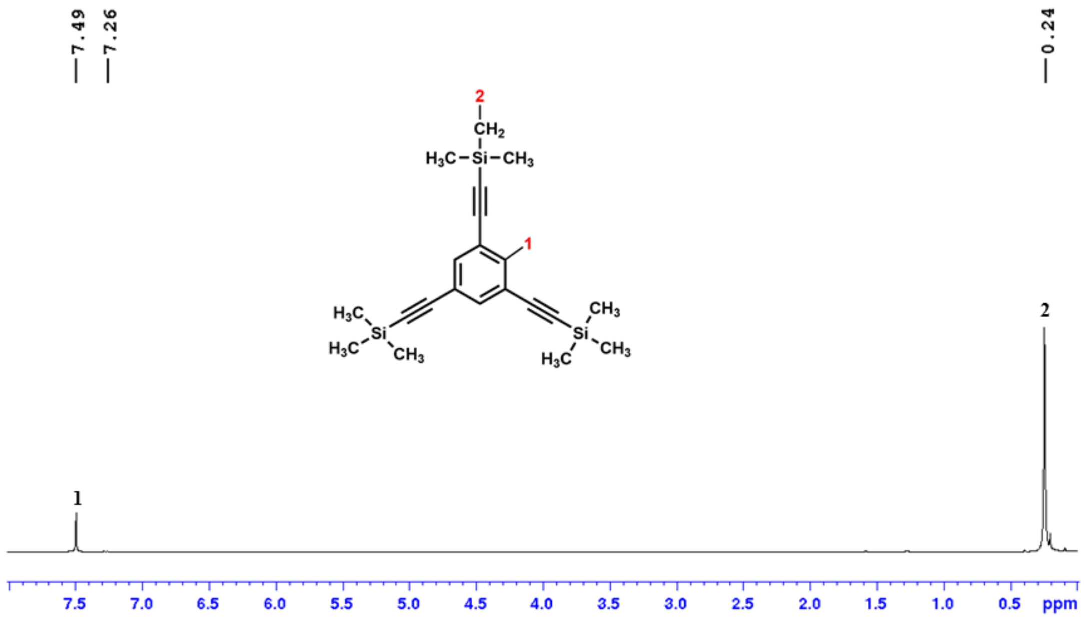

Figure S1: ^{1}H NMR of compound **A** in $CDCl_3$.

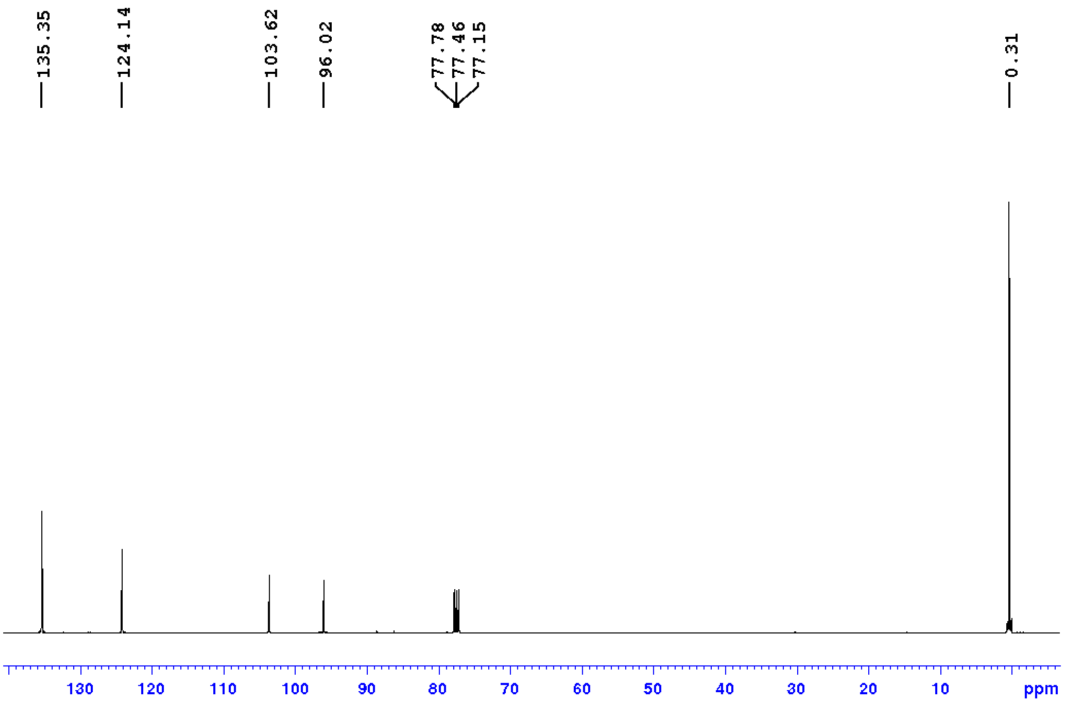

Figure S2: ^{13}C NMR of compound **A** in $CDCl_3$.

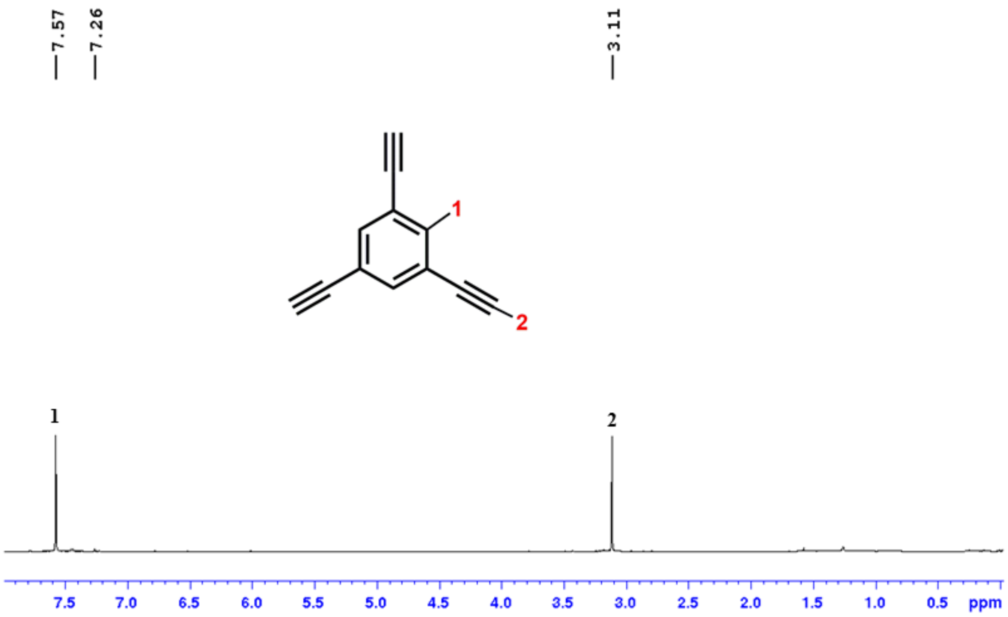

Figure S3: ^{1}H NMR of compound **B** in $CDCl_3$.

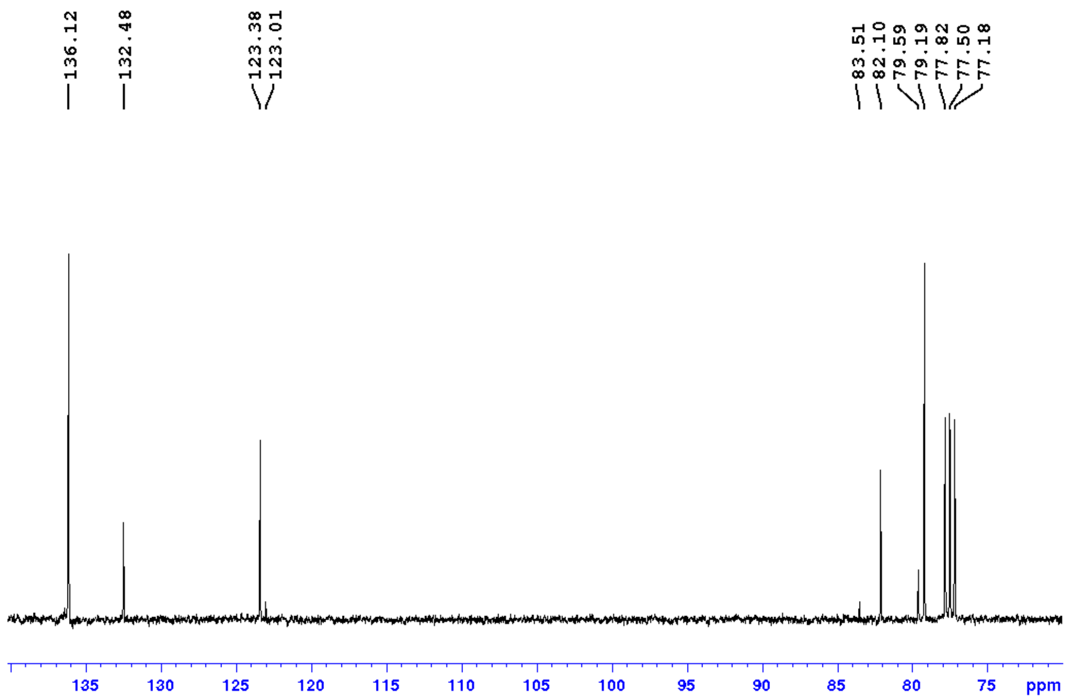

Figure S4: ^{13}C NMR of compound **B** in $CDCl_3$.

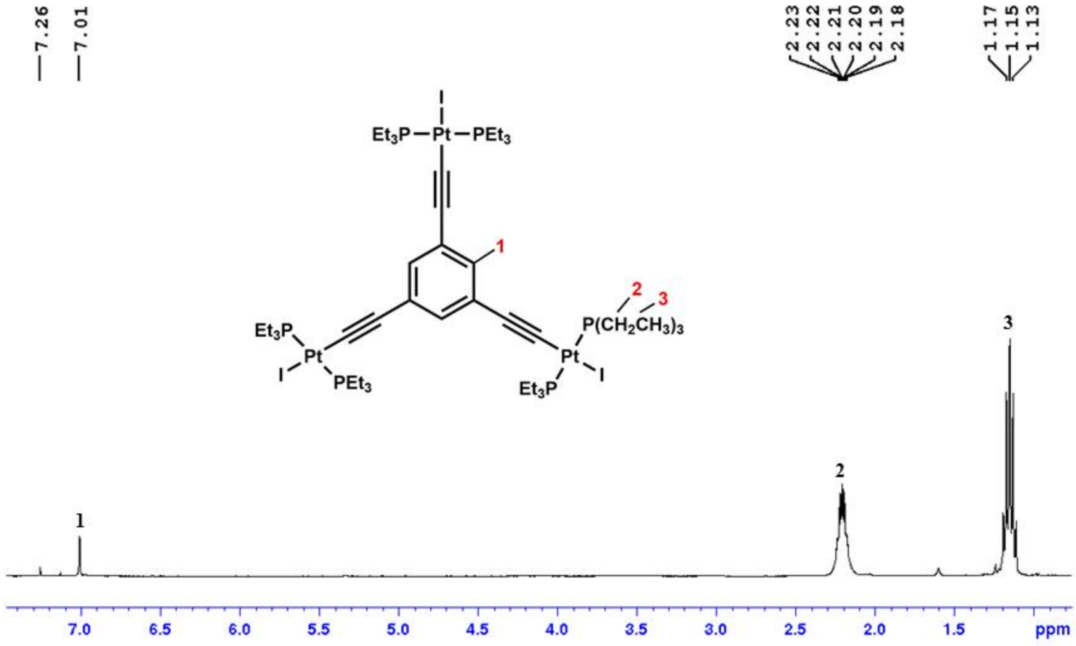

Figure S5: ^{1}H NMR of compound **C** in $CDCl_3$.

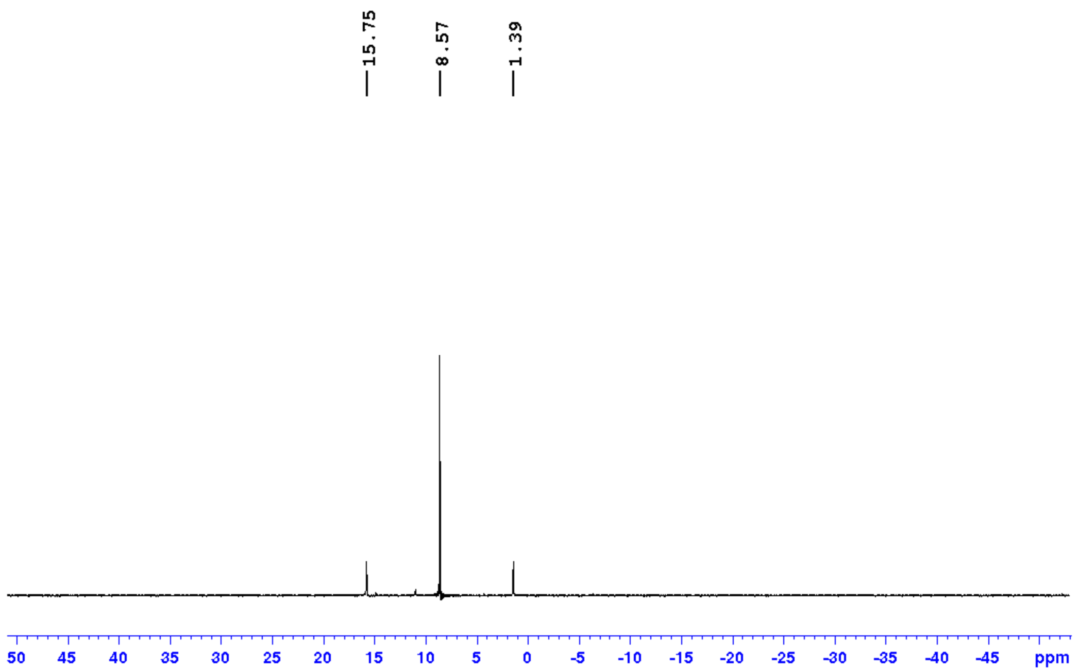

Figure S6: ^{31}P NMR of compound **C** in $CDCl_3$.

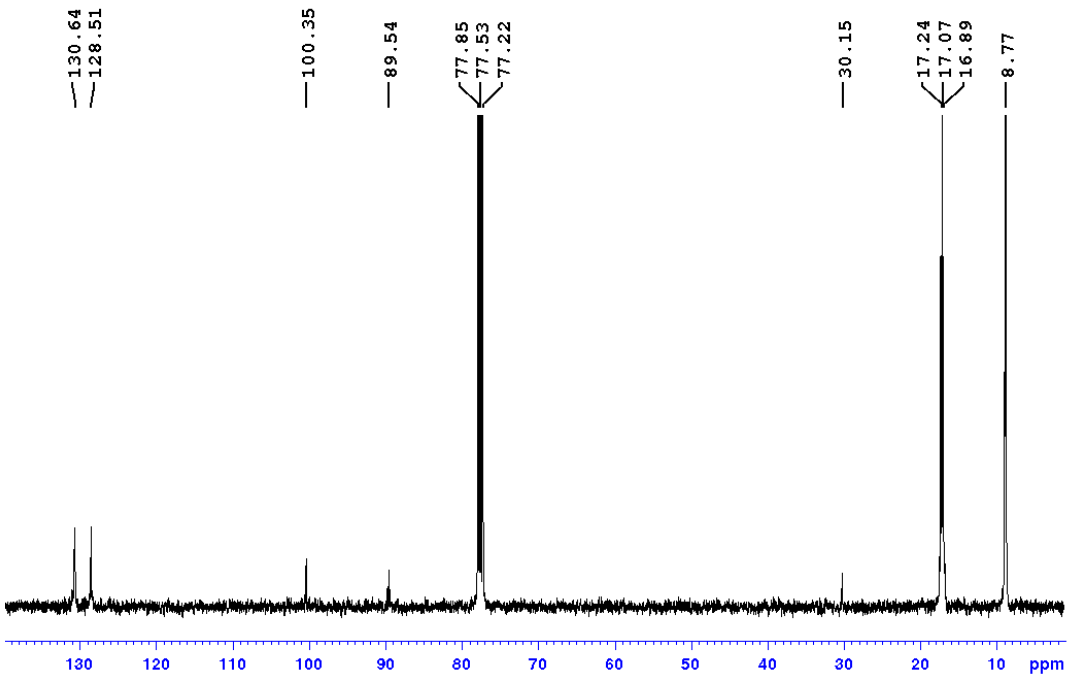

Figure S7: ^{13}C NMR of compound **C** in $CDCl_3$.

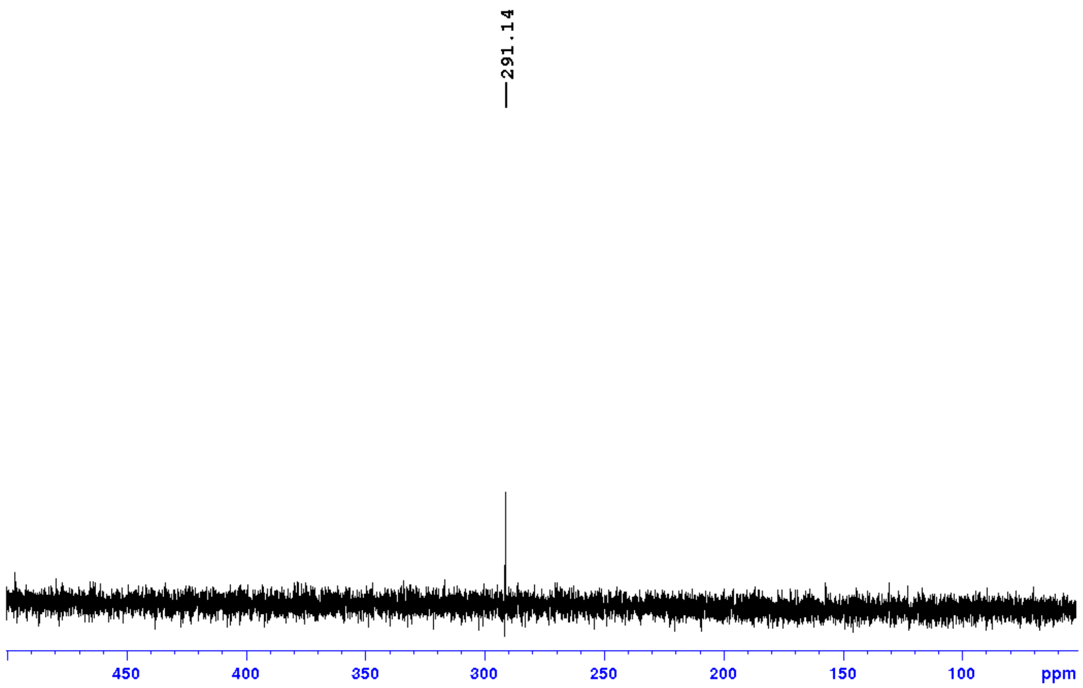

Figure S8: ^{195}Pt NMR of compound **C** in $CDCl_3$.

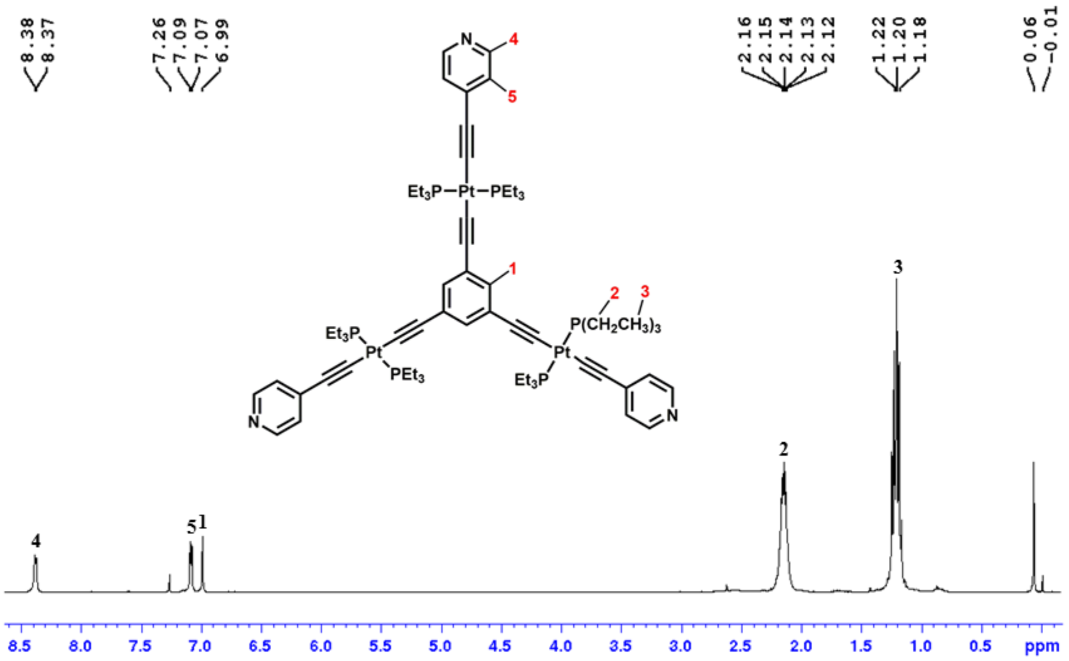

Figure S9: ^{1}H NMR of **2** in $CDCl_3$.

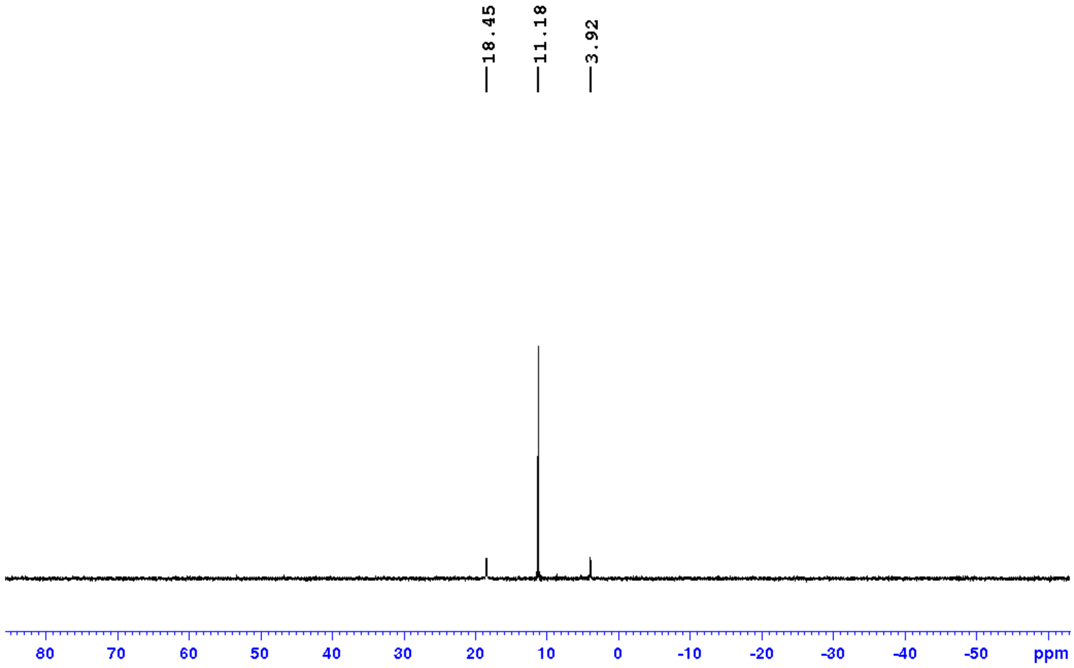

Figure S10: ^{31}P NMR of **2** in $CDCl_3$.

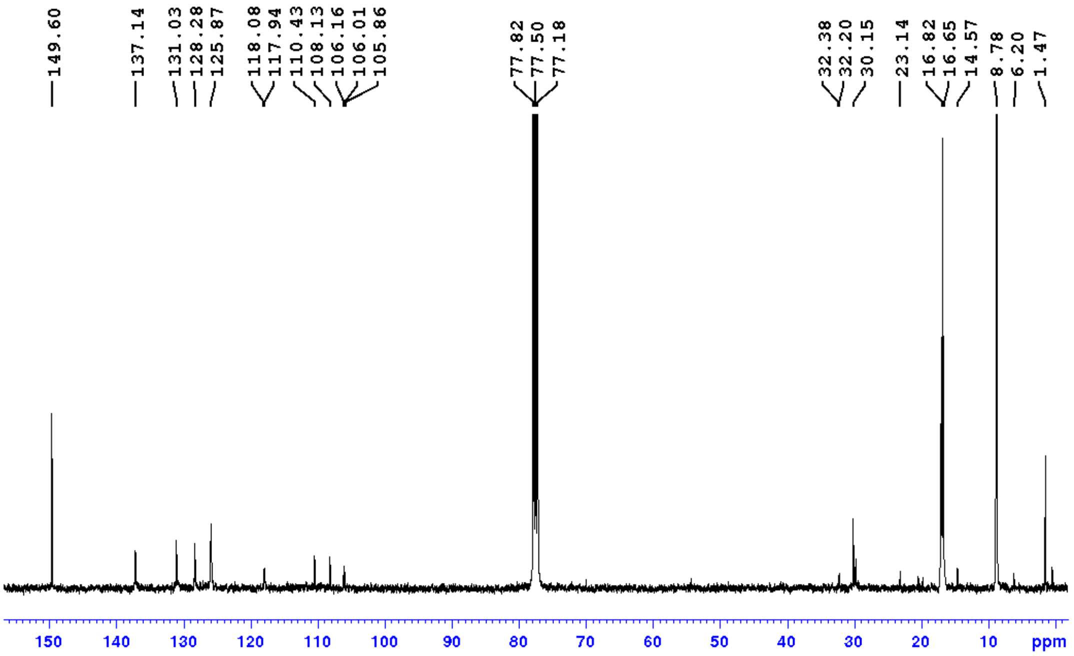

Figure S11: ^{13}C NMR of **2** in $CDCl_3$.

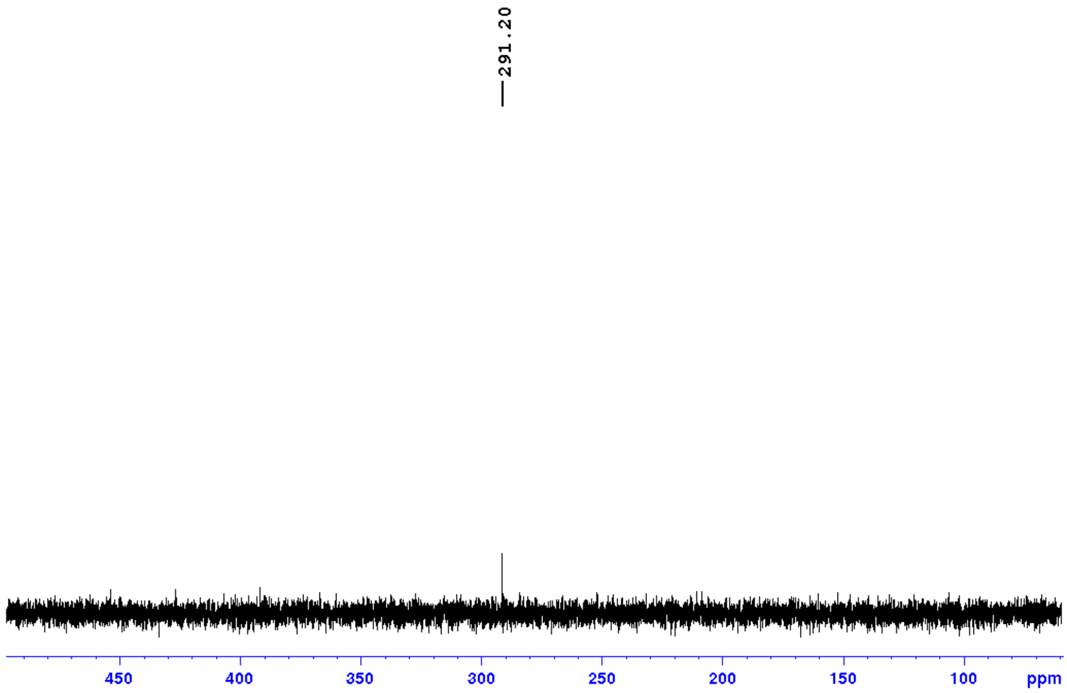

Figure S12: ^{195}Pt NMR of **2** in $CDCl_3$.

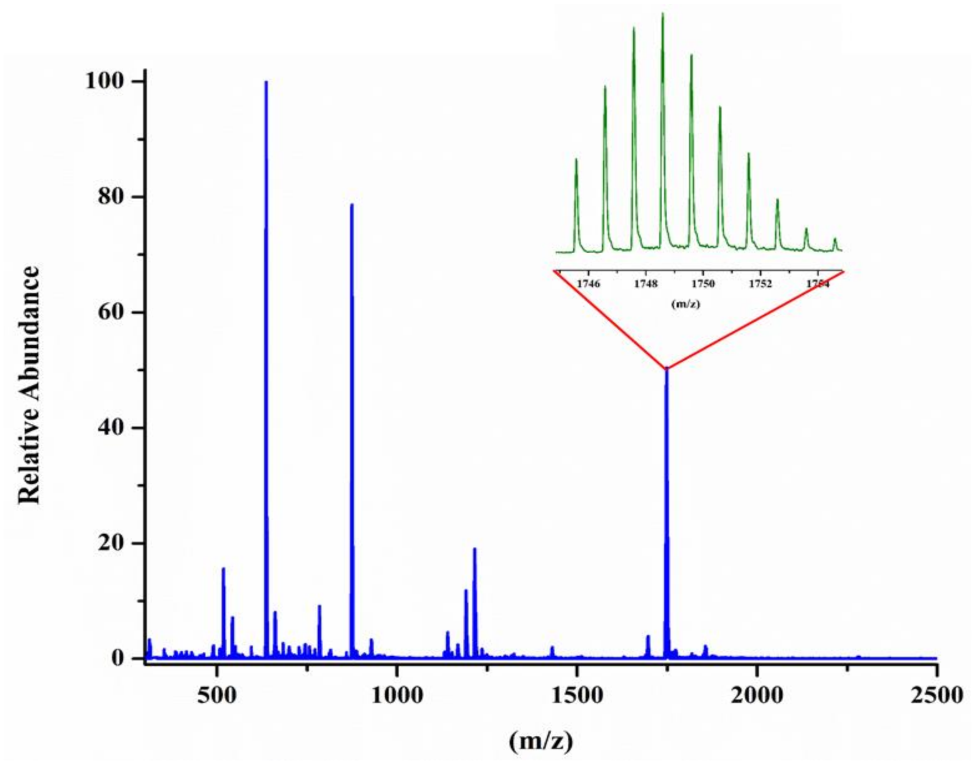

Figure S13: ESIMS spectrum of the metalloligand **2** in CH_3OH. Inset: experimentally observed isotopic distribution for [**2** + H]$^+$ fragment.

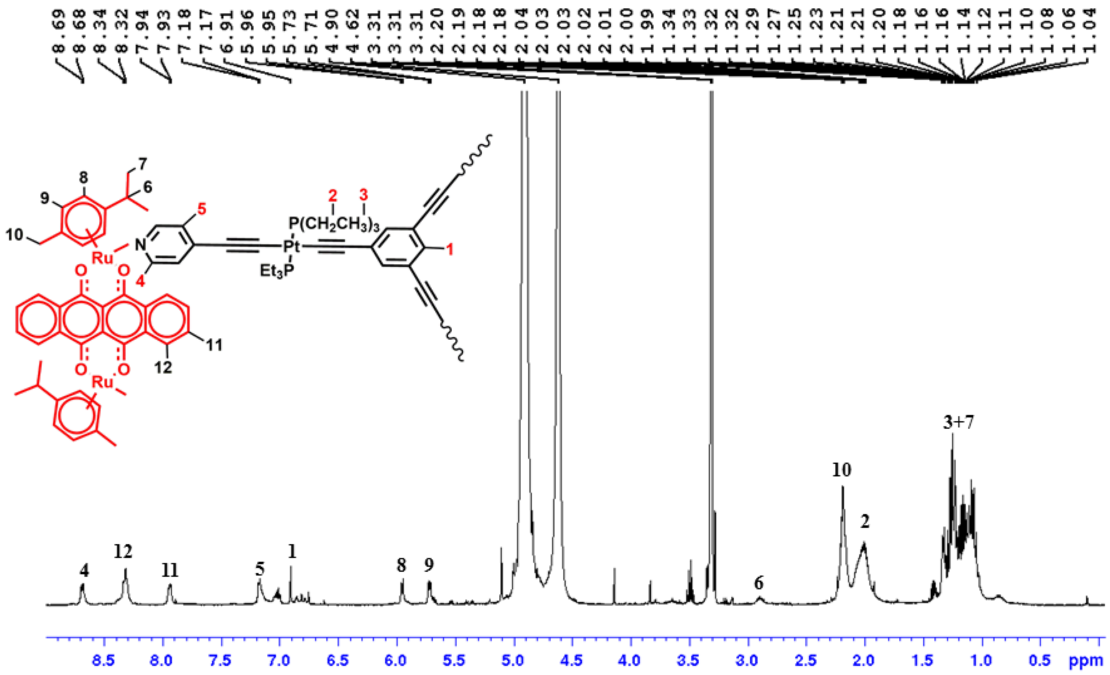

Figure S14: ^{1}H NMR of **3a** in CD_3OD.

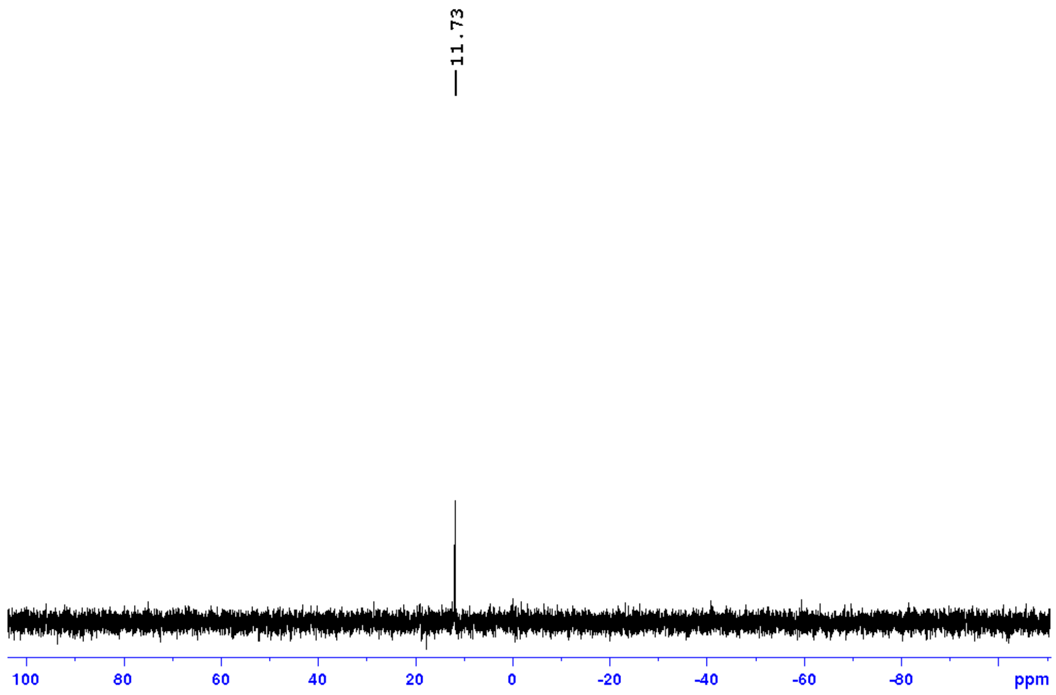

Figure S15: ^{31}P NMR of **3a** in CD_3OD.

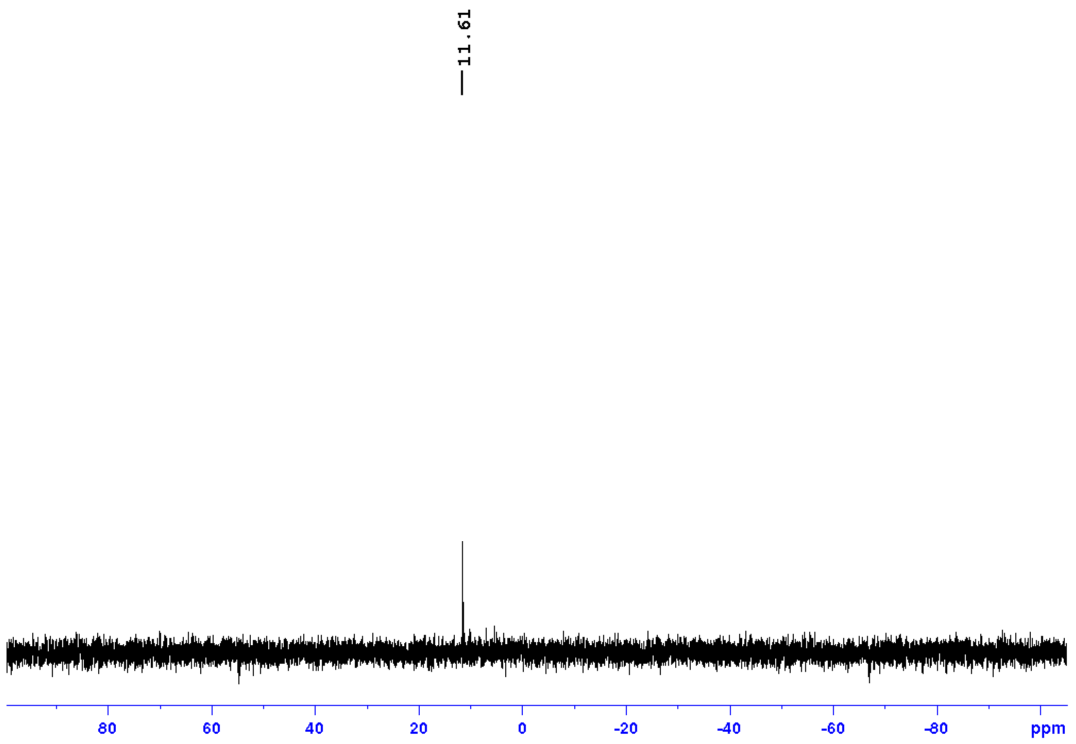

Figure S16: ^{31}P NMR of **3b** in CD_3OD.

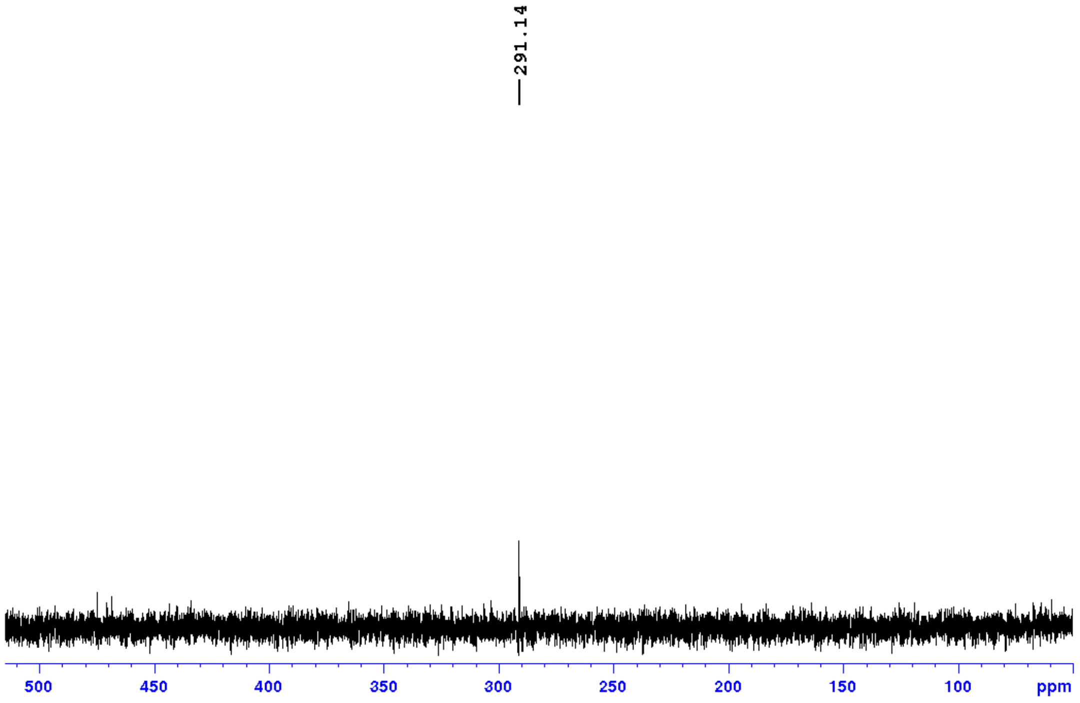

Figure S17: ^{195}Pt NMR of **3a** in CD_3OD.

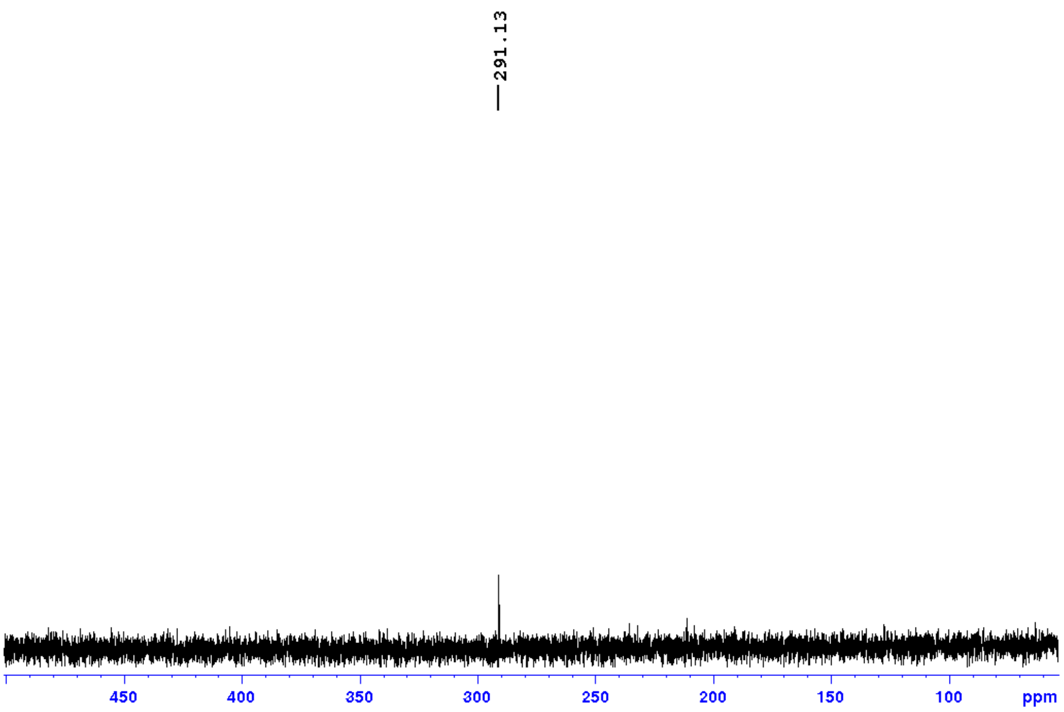

Figure S18: ^{195}Pt NMR of **3b** in CD_3OD.

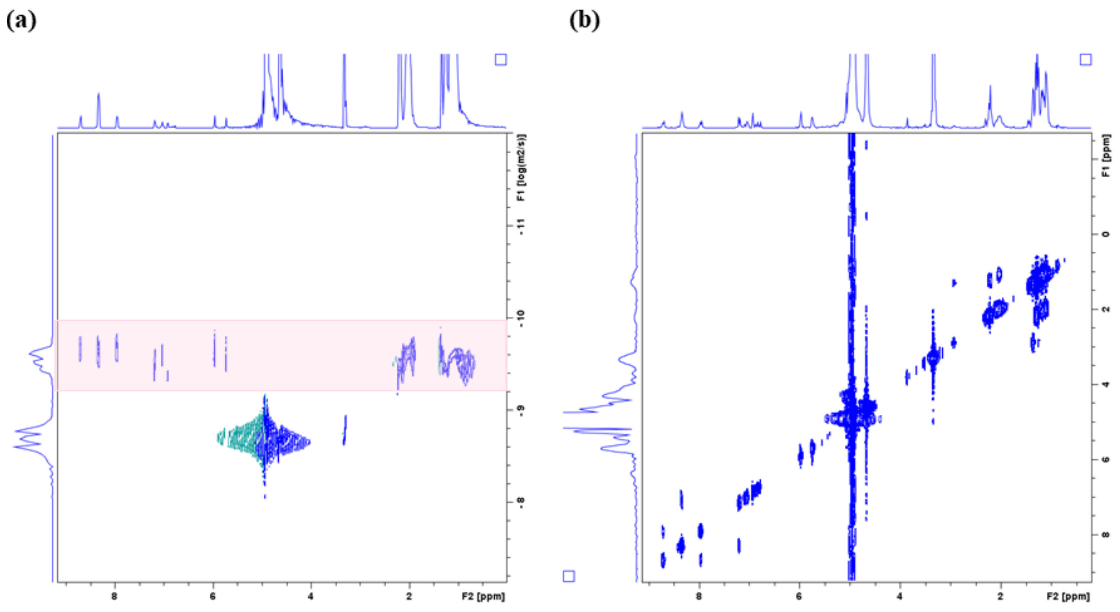

Figure S19: (a) DOSY NMR and (b) $^{1}H,^{1}H$ COSY NMR spectra of **3a** in CD_3OD.

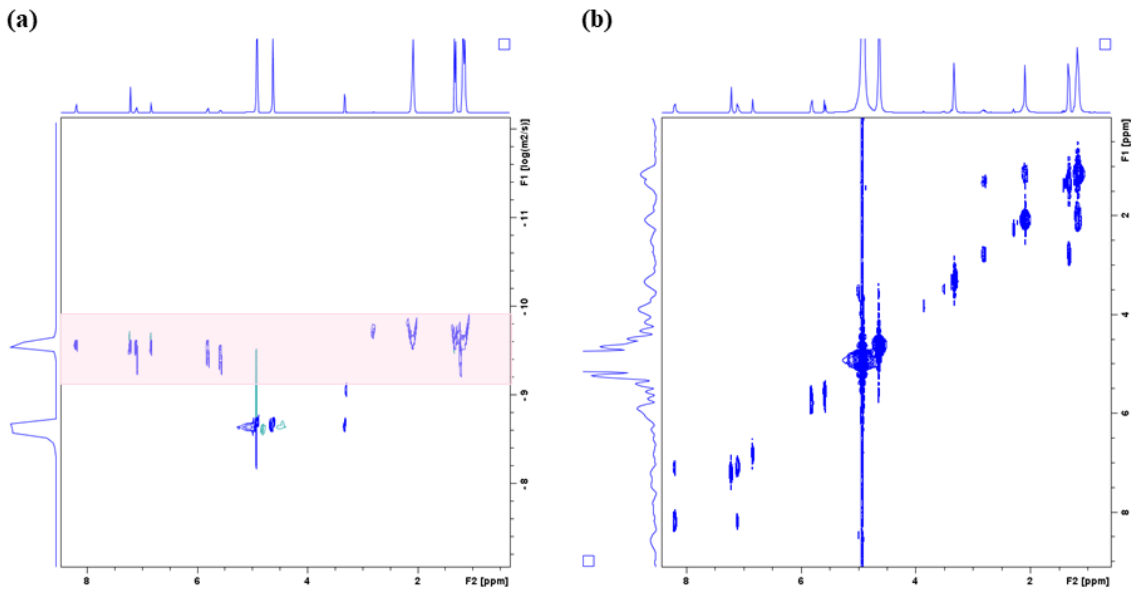

Figure S20: (a) DOSY NMR and (b) $^{1}H,^{1}H$ COSY NMR spectra of **3b** in CD_3OD.

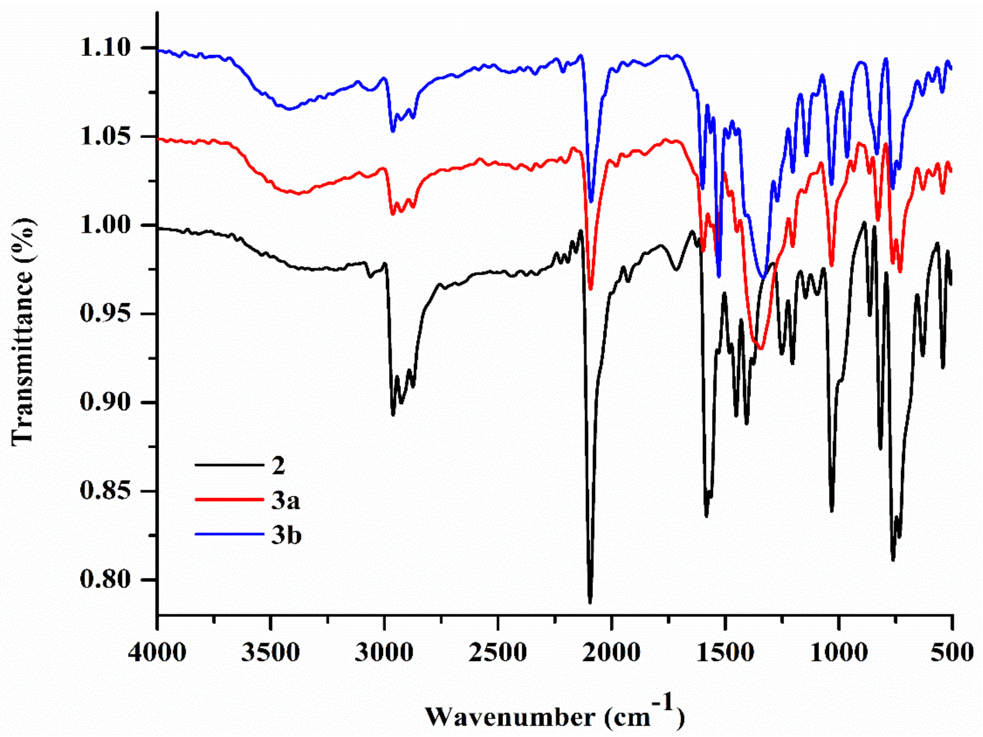

Figure S21: Infrared spectra of **2** and the heterometallic prismatic cages **3a** and **3b.**

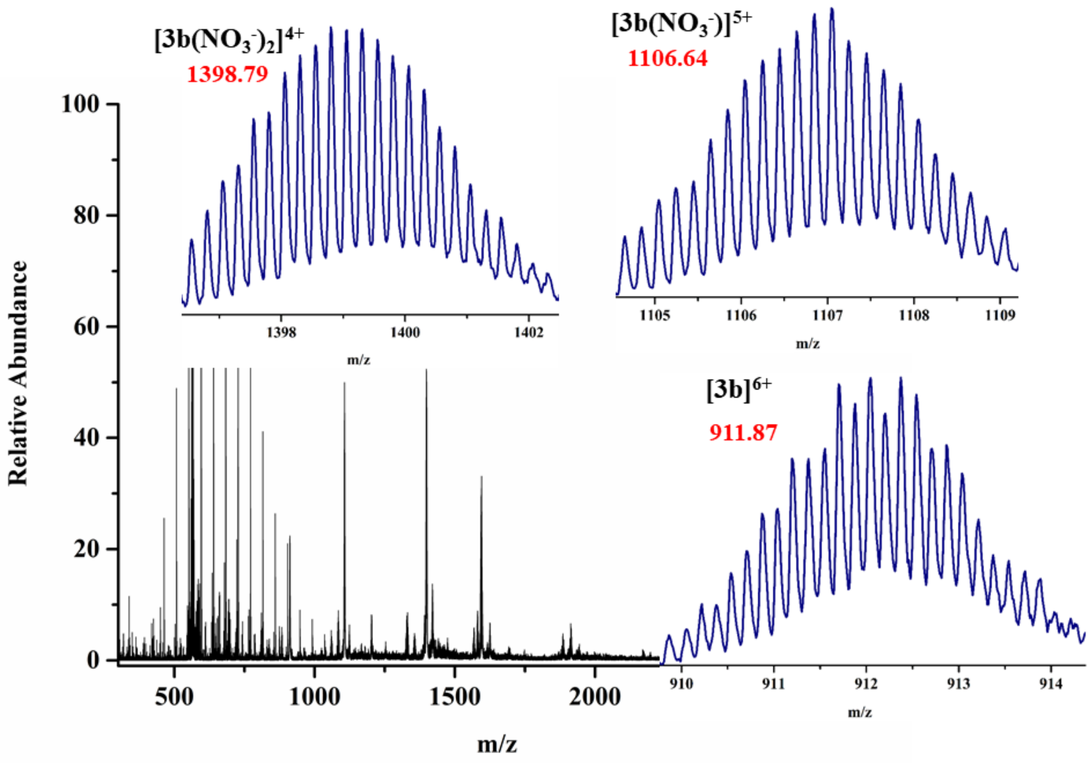

Figure S22: ESIMS spectrum of **3b** in methanol. Inset: experimentally observed isotopic distribution patterns of the charged fragments.

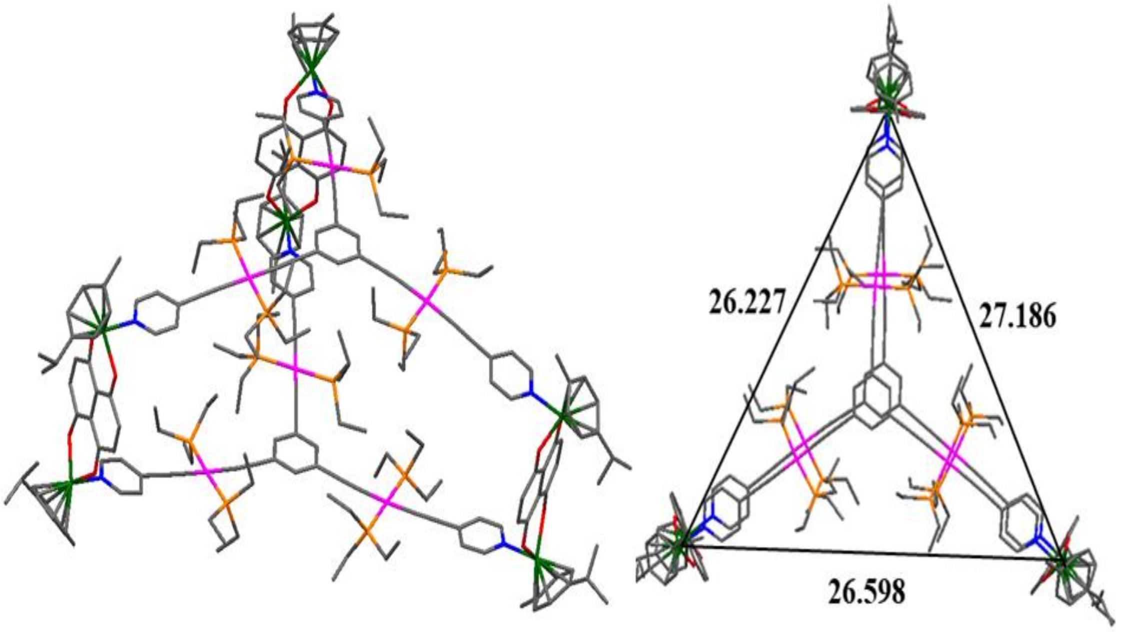

Figure S23: Energy-minimized structure of the heterometallic trigonal prismatic cage **3b**. Hydrogen atoms are omitted for the sake of clarity [Ru: green, Pt: pink, O: red, N: blue, P: orange, C: grey].

References

1. Barry, N. P. E.; Therrien, B., *Eur. J. Inorg. Chem.* **2009,** *2009* (31), 4695-4700.
2. Barry, N. P. E.; Furrer, J.; Therrien, B., *Helv. Chim. Acta* **2010,** *93* (7), 1313-1328.
